# Chemical Composition of Essential Oil from Italian Populations of *Artemisia alba* Turra (Asteraceae)

**DOI:** 10.3390/molecules170910232

**Published:** 2012-08-27

**Authors:** Antonella Maggio, Sergio Rosselli, Maurizio Bruno, Vivienne Spadaro, Francesco Maria Raimondo, Felice Senatore

**Affiliations:** 1 Department of Molecular and Biomolecular Science and Technology (STeMBio), Organic Chemistry Section, Palermo University, Viale delle Scienze, Palermo 90128, Italy; 2 Department of Environmental Biology and Biodiversity, Palermo University, Via Archirafi 38, Palermo 90123, Italy; 3 Department of Chemistry of Natural Products, University of Naples “Federico II”, Via D. Montesano, Naples 49-80131, Italy

**Keywords:** *Artemisia alba*, essential oil, biodiversity, α-bisabolone oxide A, davanone D

## Abstract

The use of essential oils as chemotaxonomic markers could be useful for the classification of *Artemisia* species and to caracterize biodiversity in the different populations. An analysis of the chemical composition of four essential oils from Italian populations of *Artemisia alba* Turra (collected in Sicily, Marche and Abruzzo) was investigated. In this paper an in depth study of the significant differences observed in the composition of these oils is reported.

## 1. Introduction

*Artemisia* L. is a large, important genus of the Asteraceae family. It comprises more than 500 species [1] although in the past this number has fluctuated depending on authors’ opinions [2,3]. *Artemisia* is a cosmopolitan genus, mainly distributed in temperate areas of mid to high latitudes of the Northern Hemisphere, with only a few representatives in the Southern Hemisphere. Central Asia is its center of diversification, while the Mediterranean region and North West America are two secondary speciation areas [4,5]. Some species are also reported in Africa and Europe [3,6].

Due to the high number of species, *Artemisia* is a taxonomically complex genus because some species have different morphological forms and others closely resemble each other. For this reason a correct identification, based only on morphological details, is quite difficult. The genus has been divided in four subgenus: *Abrotanum* Bess., *Absinthium* (Miller) DC., *Seriphidium* Bess. and *Dracunculus* Bess [7] although more recently the subgenera *Abrotanum*, *Absinthium*, *Seriphidium* have been joined in the subgenus *Artemisia* [6].

*Artemisia alba* Turra is found in the southern part of Europe and is widespread in Italy with the exception of Sardinia [8], and due to its morphological variability has an uncertain botanical placement since some authors have included it in several different subgenus: *Absinthium* [9], *Abrotanum* [10] or *Artemisia* [6]. As confirmation of this complexity, the Sicilian population of this species, due to its peculiar morphological characters, was assigned, in the past, to a differently named intraspecific taxon: *A. camphorata* Vill. var. *subcanescens* Ten. [11], *A. alba* var. *incanescens* (Jord.) Fiori [9].

Previous chemical studies indicate that patterns of secondary metabolites present in plants of the genera *Artemisia* include triterpenes, steroids, hydrocarbons, polyacetylenes, flavonoids, coumarins, mono and sesquiterpenoids with a wide range of biological activities such as antimalarial, cytotoxic, antihepatotoxic, anti-bacterial, antifungal and antioxidant properties [12,13].

Concerning phytochemical investigations of *A. alba*, only four papers have been published on the non-volatile components; three papers have been published considering its synonyms *A. lobelii* All. [14,15,16], *A. biasolettiana* Vis., *A. suavis* Jord., *A. incanescens* Jord., *A. camphorata* Vill. listed in the European Flora database [17]. Santonin was isolated from the aerial parts [18], whereas the roots were shown to contain a sesquiterpene-coumarin ether [10]. Studies on the aerial parts of *A. alba* collected in Calabria showed the absence of sesquiterpenoids and the presence of several nerolidol derivatives [19]. This latter data are in agreement with recent studies [20] according to which the population occurring in Calabria is to be assigned to a diploid subspecies (*A. alba* subsp*.chitachensis* Maire)*.* Artalbic acid, a sesquiterpene with an unusual skeleton, was isolated from the aerial parts of *A. alba* collected in Sicily [21], corresponding to a tetraploid population of this species [22].

The use of essential oils as chemotaxonomic markers could be useful for the classification of *Artemisia* species and to characterize the biodiversity of the different populations. The GC-MS analysis of essential oils of 14 *Artemisia* species collected in the North West Italian Alps has allowed us to draw some interesting considerations on the classification of the genus *Artemisia*. In particular, *A. alba* is characterized by a high content of camphor like *A. vallesiaca*, *A. glacias* and *A. vulgaris* collected in the same region [23]. Camphor and isopinocamphone were particularly high in *A. alba*. The same chemical components were found in some Belgian populations of *A. alba* [24]. The content of monoterpenic aldehydes is high too and cuminaldehyde is the second most important component in the oil [23]. An interesting paper compared the essential oil compositions of two populations of *A. alba* wild growing on calcareous and serpentine substrates and pointed out the fact that the type of soil could have an important influence on the biosynthesis of *A. alba* volatiles, especially in the case of populations grown on serpentine rock, characterized by deficiency of water and indispensable mineral elements. The camphor content is high in *A. alba* from a calcareus habitat, whereas germacrene D is the major component in serpentinophyte *A. alba* [25].

## 2. Results and Discussion

Hydrodistillation of the aerial parts of *A. alba* Turra collected in Madonie (**A**), Marche (**B**), Majella (**C**) and Mt. Velino (**D**) yielded 1.5%, 0.4%, 0.16% and 0.03% (w/w) of essential oils, respectively, all characterized by a pale yellow colour. In Table 1 the compounds identified are listed according to their retention indices on a HP-5MS column, and are classified in seven classes on the basis of their chemical structures. The composition of the oils is different, both qualitatively and quantitatively. The oil obtained from *Artemisia alba* from Madonie (**A**) is characterized by a high concentration of sesquiterpenes that represents more than 60% of the composition of the oil, while in the oils of other populations the presence of monoterpenes and sesquiterpenes is roughly equivalent.

**Table 1:** Composition (%) of essential oils from aerial parts of *Artemisia alba* Turra collected in Madonie (**A**), Marche (**B**), Majella (**C**) and Mt. Velino (**D**).

K_i_^a^	K_i_^b^	Component	Ident.	A	B	C	D
**Monoterpene Hydrocarbons**							
909	1032	Santolina triene	1, 2	7.3	1.2		
931	1023	α-Thujene	1, 2		0.1		
938	1032	α-Pinene	1, 2, 3		1.7	0.3	
953	1076	Camphene	1, 2	1.2		0.1	
973	1132	Sabinene	1, 2	0.6			
980	1118	β-Pinene	1, 2, 3	0.5	2.7	0.3	
1025	1278	*p*-Cymene	1, 2, 3	0.5		0.2	
1030	1203	Limonene	1, 2, 3	1.0			
1057	1256	γ-Terpinene	1, 2, 3	0.2			
1114	1408	1,3,8-*p*-Menthatriene	1, 2		0.1		
		**Total**		**11.3**	**5.8**	**0.9**	**-**
**Oxygenated Monoterpenes**							
1024	1402	Santolina alcohol	1, 2	2.6	0.2		
1034	1213	1,8-Cineole	1, 2, 3	1.6	0.7	0.1	
1063	1555	*cis*-Sabinene hydrate	1, 2			0.2	0.2
1063	1358	Artemisia ketone	1, 2		4.6		
1085	1512	Artemisia alcohol	1, 2		6.0		
1093	1474	*trans*-Sabinene hydrate	1, 2		1.1	0.3	t
1098	1553	Linalool	1, 2, 3		0.6		0.1
1108	1616	Hotrienol					3.3
1115	1451	β-Thujone	1, 2			0.7	
1117	1571	*trans*-*p*-Menth-2-en-1-ol	1, 2			0.3	
1125	1540	Chrysanthenone	1, 2	1.1	3.1		
1128	1487	α-Campholenal	1, 2			0.2	
1138	1664	*trans*-Pinocarveol	1, 2		0.1		
1145	1532	Camphor	1, 2, 3	1.6	3.4	0.7	
1146		Neolyratol	1, 2				0.3
1149	1685	*trans*-Verbenol	1, 2		0.2	1.8	
1164	1684	*trans*-Chrysanthenol	1, 2				0.4
1165	1587	Pinocarvone	1, 2			1.6	
1167	1719	Borneol	1, 2, 3	2.1	9.3	0.7	
1174	1565	*cis*-Pinocamphone	1, 2			14.9	1.1
1176	1611	Terpinen-4-ol	1, 2, 3	1.5	0.6		1.2
1183	1757	*cis*-Piperitol	1, 2				0.2
1185	1856	*p*-Cymen-8-ol	1, 2			0.3	0.1
1189	1706	α-Terpineol	1, 2, 3	0.5	1.2		0.7
1193	1648	Myrtenal	1, 2		0.7	0.2	
1197	1805	Myrtenol	1, 2	0.6	1.2	1.4	0.5
1201	1618	Safranal	1, 2		0.1		
1217	1845	*trans*-Carveol	1, 2	0.5	1.6	0.5	
1226	1878	*cis*-Carveol	1, 2	0.7	0.3	0.4	
1238	1694	Neral	1, 2				0.4
1241	1752	Carvone	1, 2		0.2	0.4	
1268	1741	Geranial	1, 2				0.3
1293	2198	Thymol	1, 2, 3			0.4	
1299	2239	Carvacrol	1, 2, 3		0.1		
1343	1748	Piperitone	1, 2		2.2	12.6	32.8
		**Total**		**12.8**	**37.5**	**37.7**	**41.6**
**Sesquiterpene Hydrocarbons**							
1352	1466	α-Cubebene	1, 2		0.2		
1377	1497	α-Copaene	1, 2			0.3	
1385	1535	β-Bourbonene	1, 2		0.3	0.1	
1387	1594	β-Elemene	1, 2		1.0	0.4	0.4
1415	1612	β-Caryophyllene	1, 2, 3		0.9	0.3	0.6
1437	1530	α-Guaiene	1, 2			0.2	
1453	1673	(*E*)-β-Farnesene	1, 2	1.2			
1455	1689	α-Humulene	1, 2		1.3	0.2	t
1463	1667	*allo*-Aromadendrene	1, 2	0.9			0.5
1474	1682	γ-Gurjunene	1, 2	6.4	1.0		
1477	1726	Germacrene D	1, 2		2.1	4.9	10.2
1478	1704	γ-Muurolene	1, 2		0.6		
1482	1741	β-Eudesmene (β-Selinene)	1, 2			0.3	
1486	1733	α-Selinene	1, 2		7.6	0.5	
1487	1679	α-Amorphene	1, 2				0.4
1489	1729	(*Z,E*)-α-Farnesene	1, 2	2.7			
1490	1694	β-Guaiene	1, 2	0.2	0.3		
1491	1756	Bicyclogermacrene	1, 2		2.5		
1506	1760	(*E,E*)-α-Farnesene	1, 2	1.5			
1509	1746	*cis*-(*Z*)-α-Bisabolene	1, 2	0.8	2.7		
1510	1743	β-Bisabolene	1, 2				0.6
1515	1776	γ-Cadinene	1, 2	0.5			
1520	1839	1-S-*cis*-Calamenene	1, 2		0.1		
1526	1773	δ-Cadinene	1, 2	1.5	0.4	0.3	0.4
1554	1856	Germacrene B	1, 2			0.3	
		**Total**		**15.7**	**21.0**	**7.8**	**13.1**
**Oxygenated Sesquiterpenes**							
1234	1641	*nor-*Davanone	1, 2	0.1			
1457	1712	Cabreuva oxide B	1, 2				0.9
1476		Davana ether	1, 2	0.3			
1534	1991	Artedouglasia oxide A	1, 2	0.9			
1559	1967	Davanone B	1, 2	0.8			
1563	2065	Artedouglasia oxide D	1, 2	0.6			
1564	2050	(*E*)-Nerolidol	1, 2			0.6	6.4
1564	2056	Ledol	1, 2		0.2		
1578	2150	Spathulenol	1, 2, 3	1.6	4.2	0.4	2.1
1580	2008	Caryophyllene oxide	1, 2, 3		1.8	1.1	2.0
1587	2108	Dihydronerolidol	1,2				2.9
1588	2025	Davanone D	1, 2	10.5			
1591	2104	Viridiflorol	1, 2		0.4		
1598	2107	Guaiol	1, 2		2.8		
1638	2223	Isospathulenol	1, 2	0.7	0.1		
1640	2185	T-Cadinol	1, 2				2.8
1641	2209	T-Muurolol	1, 2				0.9
1648	2399	Aromadendrene oxide	1, 2				1.0
1653	2252	α-Eudesmol	1, 2			42.2	
1655		a C_15_H_22_O	1, 2		1.1		
1657	2217	α-Bisabolone oxide A	1, 2	16.4			
1658	2156	α-Bisabolol oxide B	1, 2	2.2			
1675	2213	(*Z*)-α-Bisabolene epoxide	1, 2	0.5			
1682	2246	Bisabolone oxide	1, 2	9.0			
1682	2232	α-Bisabolol	1, 2	0.8	1.7		4.5
1687	1896	*allo*-Aromadendrene oxide	1, 2		0.4	0.2	0.8
1689	2359	8-Cedren-13-ol	1, 2		10.3		
1692	2342	(2*Z*,6*E*)-Farnesol	1, 2				1.9
1692	2245	*epi*-α-Bisabolol	1, 2	0.8			4.7
1738	2162	α-Bisabolol oxide A	1, 2	1.4			
1765	2518	*cis*-Lanceol	1, 2	0.4	0.2		
		**Total**		**47.0**	**23.2**	**44.5**	**30.9**
**Others**							
977	1452	1-Octen-3-ol	1, 2			0.2	
1123	1570	Isophorone	1, 2		t		
1206	1510	Decanal	1, 2		0.2		
1397	1959	*cis*-Jasmone	1, 2	0.1			
1405	2031	Methyleugenol	1, 2	0.6	0.3		
		**Total**		**0.7**	**0.5**	**0.2**	**-**
**Esters**							
1235	1583	*trans*-Chrysanthenyl acetate	1, 2	1.4	2.1		1.6
1241		Linalyl formate	1, 2		0.1		
1264	1561	*cis*-Chrysanthenyl acetate	1, 2	0.6	t		1.5
1286	1567	Bornyl acetate	1, 2, 3	0.5		0.2	
1325	1678	Myrtenyl acetate	1, 2			0.3	
1362	1729	Neryl acetate	1, 2				0.5
1818	1716	(2*Z*,6*E*)-Farnesyl acetate	1, 2	t			
		**Total**		**2.5**	**2.2**	**0.5**	**3.6**
**Oxygenated diterpenes**							
2135	2625	(*E*)-Phytol	1, 2				1.1
		**Total compounds**		**47**	**57**	**41**	**38**
		**TOTAL**		**90.0**	**90.2**	**91.6**	**90.3**

^a^: Ki = Kovats index; HP-5 MS column; ^b^: Ki = Kovats index; HP Innowax column; 1: retention index, 2: mass spectrum, 3: co-injection with authentic; compound t: traces, less than 0.05%.

All oil extracts from the populations of Marche, Majella and Monte Velino have a content of monoterpenes (43.3%, 38.6% and 41.6%, respectively), which is about twice as high compared with the same class of compounds identified in the oil from Madonie (24.1%).

Among the monoterpenic hydrocarbons in the oil from Madonie, santolinatriene (7.3%), an irregular monoterpene, predominates and it is also present in low concentrations in **B**, but absent in **C** and **D**. On the other hand in the oil from Marche irregular oxygenated monoterpenes are found in higher concentrations. In fact, santolina alcohol, artemisia alcohol, artemisia ketone and chrysanthenone represent about one third (13.9%) of the fraction while in the oil from Madonie santolina alcohol, despite being the most abundant oxygenated monoterpene, accounts for only 2.6%, the remaining (10.2%) of this fraction being constituted by regular oxygenated monoterpenes. The most abundant oxygenated monoterpenes of oil from Marche are borneol (9.3%), artemisia alcohol (6.0%) and artemisia ketone (4.6%); the last two being absent in **A**, **C** and **D**. In the oils from Abruzzo (Majella, **C** and Monte Velino, **D**) monoterpenic ketones (*cis*-pinocamphone, piperitone) are prevalent instead and they account for more than half of the content of monoterpenes.

Concerning the content of oxygenated sequiterpenes, although the total percentages are similar in the four populations, the proportion of the various types of compounds changes drastically. In fact in **A** ketones (11.5%) and oxides (31.4%) are prevalent with davanone D (10.5%) and α-bisabolone oxide A (16.4%) as main compounds, while alcohols represent only 4.3%. On the other hand in **B**, **C** and **D**, the content of sesquiterpene alcohols is very high (19.9%, 42.2% and 26.2%, respectively). The main compounds among the sesquiterpene alcohols are: 8-cedren-13-ol (10.3%) in the oil from Marche, α-eudesmol (42.2%) in the oil from Majella and *epi*-α-bisabolol (4.7%), α-bisabolol (4.5%) and (*E*)-nerolidol (6.4%) in the oil from Monte Velino.

According to the literature [17] α-thujone and camphor are two markers allowing a distinction of *Artemisia* in two groups. Our four oils are characterized by the absence of α-thujone, whereas camphor and its biogenetic precursor, borneol are present in **A**, **B** and **C**.

## 3. Experimental

### 3.1. Plant Material

**Figure 1:**
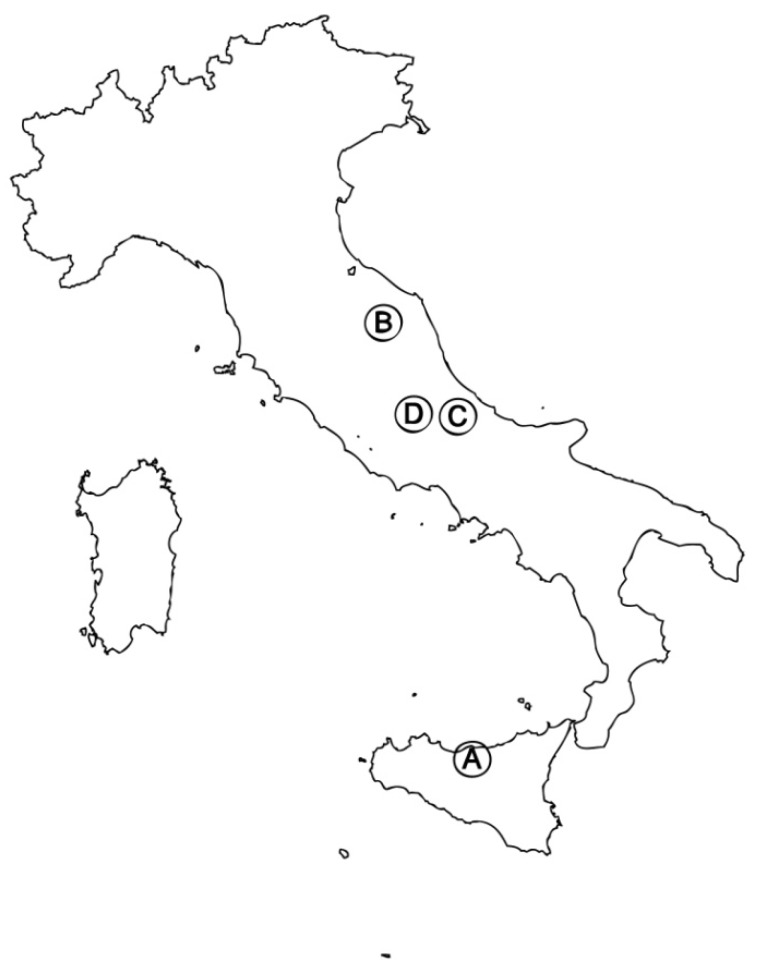
Map of the samples’ origins: Madonie (**A**), Marche (**B**), Majella (**C**) and Mt. Velino (**D**) are indicated.

The aerial parts of the four populations of *Artemisia alba* Turra, were collected from blooming plants in Sicily, pastures on carbonate soils at Pizzo Carbonara (Madonie), in spring of 2011 (**A**); Marche, pastures on carbonate soils between Fabriano (Ancona) and Matelica (Macerata), in spring of 2011, Abruzzo: pastures on carbonate soils at Mt Majella (**C**) and Mt Velino (**D**), in summer of 2011 (Figure 1). Samples of the studied material, identified by the authors F. M. Raimondo and V. Spadaro, are kept in the Herbarium Mediterraneum of the Palermo University [Raimondo & Spadaro (PAL)].

### 3.2. Isolation of the Essential Oil

The air-dried samples were ground in a Waring blender and then subjected to hydrodistillation for 3 h using *n*-hexane as solvent, according to the standard procedure of the *European Pharmacopoeia* [26]. The extracts were dried over anhydrous sodium sulphate and then stored in sealed vials, at −20 °C, ready for the GC and GC-MS analyses. The samples yielded 1.5% (**A**), 0.40% (**B**), 0.16% (**C**) and 0.03% (**D**) (w/w) of pleasant smelling yellow oils.

### 3.3. Gas Chromatography-Mass Spectrometry

Analytical gas chromatography was carried out on a Perkin-Elmer Sigma 115 gas chromatograph (Napoli, Italy) equipped with a HP-5MS capillary column (30 m × 0.25 mm, 0.25 μm film thickness), a split-splitless injector heated at 250 °C and a flame ionization detector (FID) at 280 °C. Column temperature was initially kept at 40 °C for 5 min, then gradually increased to 250 °C at 2 °C/min, held for 15 min and finally raised to 270 °C at 10 °C/min. The injection volume was 1.0 μL (split ratio 1:20). A fused silica HP Innowax polyethylene glycol capillary column (50 m × 0.20 mm, 0.25 μm film thickness) was also used for analysis. In both cases helium was the carrier gas (1 mL/min). GC-MS analysis was performed on an Agilent 6850 Ser. II apparatus (Napoli, Italy), fitted with a fused silica DB-5 capillary column (30 m × 0.25 mm, 0.33 μm film thickness), coupled to an Agilent Mass Selective Detector MSD 5973; ionization voltage 70 eV; electron multiplier energy 2000 V; source temperature 250 °C. Mass spectra were scanned in the range 35–450 amu, scan time 5 scans/s. Gas chromatographic conditions were the same as those for GC; transfer line temperature, 295 °C.

### 3.4. Identification of Components

Most of the constituents were identified by GC by comparison of their retention indices (K_i_) with either those in the literature [27,28] or with those of authentic compounds available in our laboratories. Retention indices were determined in relation to a homologous series of *n*-alkanes (C_8_–C_28_) under the same conditions. Whenever possible, co-injection with authentic substances was also performed. Component-related concentrations were calculated based on GC peak areas without using correction factors. Further identification of oil components was achieved by comparing their mass spectra on both columns, either with those stored in NIST 02 and Wiley 275 libraries or with mass spectra from the literature [28,29] and our personal library.

## 4. Conclusions

The differences in composition between the four oils makes it possible to hypothesize that the Italian populations of *Artemisia alba* Turra growing on the Madonie (Sicily), in the Marche region, on the Majella and Monte Velino (Abruzzo)—in part related to different cytotypes [19]—surely express from climatic as well as genetic differences. Furthemore, the differences of the oil of the population of the *Artemisia alba* Turra from Madonie—the most southerly of the species—let us consider that this belongs to a different chemotype from the other ones.
